# Effect of Salt on Synthetic Cationic Antimicrobial Polymer–Cell Interactions

**DOI:** 10.1021/acs.biomac.4c01706

**Published:** 2025-05-19

**Authors:** Zachary Benmamoun, Thomas Kinard, Prem Chandar, Joe Jankolovits, William A. Ducker

**Affiliations:** † Department of Chemical Engineering, 1757 Virginia Tech, Blacksburg, Virginia 24060, United States; ‡ Unilever Research & Development, Trumbull, Connecticut 06611, United States

## Abstract

Cationic antiseptics are deployed in a variety of settings, where salinity ranges from almost pure water to hypertonic salt. Here, we examine how dissolved NaCl affects the antimicrobial action of a model antimicrobial, polydiallyldimethylammonium chloride (PDADMAC) to the bacterium Escherichia coli (E. coli). Fluorescence microscopy is used to measure the time course of both the adsorption of PDADMAC to E. coli and the cell viability. NaCl decreases the density of adsorbed PDADMAC and diminishes its efficacy. At NaCl concentrations at or above 0.15 M, PDADMAC no longer kills bacteria but still prevents reproduction by halting the growth in cell length. Reproduction can be restarted if PDADMAC is removed. Fluorescence depolarization measurements show that PDADMAC rigidifies model membranes, but salt reduces the rigidity. We therefore attribute the halt in cell growth to reversible bridging by the polymer on the cell surface that prevents expansion of the cell membrane.

## Graphical abstract

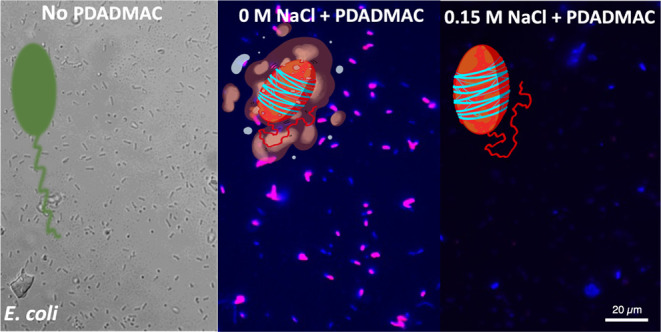

## Introduction

Synthetic polycations are positively charged polymers that are often antimicrobial^1–4^ and can be used for surface disinfection, such as on hard surfaces, skin, or textiles, and in solution. This contrasts with medicinal antibiotics that can be used to treat infections on or inside the body. Polycationic antimicrobials need to be deployable in a wide range of environments, so it is of interest to study the antimicrobial action as a function of solution condition. Here, we focus on the effect of ionic strength, which is important for two reasons: (1) salts are known to affect the behavior of polycations, and (2) salts are commonly found where antimicrobials are used. Some salt is found in most biological fluids, occurs in the ocean and natural water bodies, and is added to pools, hot tubs, and fountains. Salts are also found on inert surfaces and on skin, where polycationics may be deployed.

Salts are known to reduce the radius of gyration of polyelectrolytes in solution,^5^ alter the structure of adsorbed polyelectrolytes,^6^ and alter their density of adsorption to inorganic surfaces.^7^ Salt can have two related effects on electrostatic forces: (1) by binding to charges on polymer and membrane lipid, salt can change the magnitude of effective charge, and (2) salt can screen electrostatic interactions. Both reduce the magnitude of electrostatic forces in monovalent salts. For example, addition of salt to tethered cationic polyelectrolytes reduces the polymer layer thickness.^3^ On the other hand, when salt reduces the radius of gyration of polyelectrolytes in solution, this can lead to thicker layers that are assembled from solution in layer-by-layer assembly.^8,9^ Salt also has been shown to reverse binding of polyelectrolytes to liposomes.^10^

Our focus here is on synthetic antimicrobials, but several groups have studied the effect of ionic strength on natural cationic antimicrobial molecules that are sometimes for internal use.^11,12^ Salt generally inhibits the efficacy of natural cationic antimicrobials.^13–16^ Some antimicrobial peptides, such as spheniscins, are more resistant to the effects of salt, and this was attributed to the high density of cations.^16^ Antimicrobial peptides with added conformational rigidity have been synthesized and were found to retain antimicrobial resistance in salt solutions.^17–19^ Additionally, several researchers have examined the effect of alkylating cationic polymers on antimicrobial activity.^20,21^ In general, some degree of alkylation increases the antimicrobial activity, but some have found 6–12 methylene units is the optimal length of the alkyl chain.^22,23^

Here, we examine the effect of NaCl on polydiallyldimethylammonium chloride (PDADMAC) antimicrobial activity against a Gram-negative bacterium, Escherichia coli (E. coli). E. coli is a common environmental and enteric organism that is transmitted by a variety of mechanisms.^24^ PDADMAC is a known antimicrobial polycation and is well-studied.^25–27^ Here, we examine a range of NaCl concentrations but focus on 0.15 M NaCl (M represents molarity) because it has a similar NaCl concentration and ionic strength to normal saline. We focus on 4 × 10^5^ g mol^–1^ PDADMAC because its antimicrobial activity in salt-free solutions was examined in prior work.^28^ Adamczyk et al. measured the effect of NaCl on the solution properties of PDADMAC (1 × 10^5^ g mol^–1^) and found that, from 10^–4^ M NaCl to 0.15 M NaCl, the fraction of charged monomers dropped from 13% to 8%, and the equivalent cylinder length decreased from 240 to 72 nm.^29^ E. coli is negatively charged in water and salt solution,^30^ and therefore PDADMAC adsorbs strongly to E. coli. Previous research showed that (1) PDADMAC permeabilizes bacterial cell membranes only after a threshold adsorption has been reached and (2) there is considerable diversity among cell responses.^28^ Here, we show that 4 × 10^5^ g mol^–1^ PDADMAC in 0.15 M NaCl no longer kills E. coli, but, interestingly, the cells are prevented from growing. By preventing growth, colony formation is inhibited. Salt also decreases the polymer density on the cell surface, which is likely the cause of the decrease in antimicrobial activity, and salt makes polymer adsorption reversible.

## Materials and Methods

### Materials

Labeled PDADMAC was purchased from Surflay Nanotec (Berlin, Germany) with, on average, one in every 196 monomers containing the Cy3 fluorophore. The polymer was synthesized by copolymerization of labeled and unlabeled monomers. The molecular weight of all polymers was measured by the combination of size exclusion chromatography to separate the different molecular weights, followed by matrix-assisted laser desorption ionization (MALDI) mass spectrometry. The labeled polymer showed a bimodal molecular weight distribution with number-average modes of 3.0 × 10^5^ and 4.6 × 10^5^ g/mol, and polydispersity indexes of 3.7 and 1.2, respectively. Unlabeled PDADMAC samples that were nominally 8500 and 250,000 g/mol were purchased from Polysciences (Warrington, PA). The measured number-average molecular weights were 9.3 × 10^3^ and 4.4 × 10^4^ g/mol, and the polydispersity indexes were 1.7 and 2.3, respectively. For the rest of this article, they will be referred to by their measured molecular masses. Sytox Blue Dead Cell Stain was purchased from Fisher Scientific. CdSe/ZnS core–shell quantum dots stabilized with octadecylamine ligands were purchased from Millipore Sigma. 1,6-Diphenyl-1,3,5-hexatriene (≥98%) (DPH) was purchased from Cayman Chemical Co. (Ann Arbor, MI, USA). l-α-Phosphatidylglycerol (egg, chicken) (sodium salt) (>99%) (egg PG) was purchased from Avanti Polar Lipids (Alabaster, AL, USA). Sodium chloride (NaCl) and poly­(vinyl alcohol) (Mowiol 4-98) (PVA) were purchased from Sigma-Aldrich (St. Louis, MO, USA). Tryptic soy broth (TSB) and tryptic soy agar (TSA) were purchased from BD (Sparks, MD, USA).

### Growth of Microbial Strains

Frozen stocks of ATCC 25922 E. coli and ATCC 6538 Staphylococcus aureus (S. aureus) were streaked onto TSA plates and incubated for 24 h at 37 °C. A single colony was selected for each experiment to obtain a monoclonal culture. Cells from the selected colony were grown in suspension to stationary phase in 10 mL of TSB for 48 h at 37 °C with aeration (200 rpm). The cell purity of the suspension was checked by streaking onto a TSA plate and inspecting the grown colonies after 24 h of incubation at 37 °C. For colony-forming unit (CFU) assays, the cell suspension was adjusted to 3 × 10^8^ CFU/mL and resuspended in deionized water or a buffer solution. For flow cell experiments, the cell suspension was used directly.

### Flow Cell

Flow cell experiments were performed as previously described.^28^ Briefly, parallel-plate flow chambers were constructed from polycarbonate walls and cover-glass windows. Quantum dots (QDs) were adsorbed to the interior surface to serve as a fluorescence intensity standard in all experiments except for the 0.3 and 0.5 M salt experiments reported in Figure 3. A bacterial suspension was injected into the channels and allowed to adsorb onto the flow window. An upright microscope was used; therefore, observation was at the upper wall of the flow cell. To obtain more cells for observation, the flow cell was inverted for 20 min at the start of the experiment. Unadhered bacteria were removed by TSB flow, and then a PDADMAC and SYTOX Blue solution was flowed over the cells at 4 mL/h. Flow continued for the duration of the experiment.

### Imaging

Time-lapse images were recorded at 30 s intervals using a Zeiss Imager.M2 fluorescence microscope with a 63× objective. Three images were taken for each time point: a phase-contrast channel to view the cells and determine cell size, a fluorescence channel (550 nm excitation/605 nm emission, 20 ms exposure) to view labeled polymer (PDADMAC), and a second fluorescence channel (390 nm excitation/460 nm emission, 20 ms exposure) to image the SYTOX Blue permeability stain. All of the color in the images is false. The light exposure times were very short compared to the period between images; therefore, the three images were effectively simultaneous. For all images in this paper, SYTOX Blue was colored red for consistency, with the reader’s experience of dead stains (e.g., propidium iodide) being colored red.

The time-lapse images were thresholded. Trackpy (https://github.com/soft-matter/trackpy), a Python package, was used to identify and track cells, and then custom Python code was used to obtain the brightness, size, particle number, and location of each cell for further analysis. Cell brightness (signal level for each pixel) was divided by the square of the cell size to obtain intensity. So that measurements were comparable over time and between experiments, we added quantum dots as reference intensity standards. Intensity was normalized by the average quantum dot intensity in the same field of view at the same time.

### Cell Suspension Density

Cell suspension density was measured as CFU/mL. A CFU count is the number of cells that grow into colonies (a “viable cell” count). For CFU measurements, 10 μL of a bacterial cell suspension was mixed with 90 μL of a test solution. A 10-fold dilution series was prepared for each mixture; 100 μL of each target dilution was spread on TSA plates in triplicate, and colonies were counted after 24 h of incubation at 37 °C. Residuals were approximately normal after a log transform, so all CFU statistics were calculated from log CFU. If no colonies grew for the least dilution (the result was below the detection limit), then the CFU was set to one to enable the log transform.

### Measurement of Fluorescence Anisotropy in Model Membranes

Model membranes were produced using egg PG, which was employed to mimic negatively charged and compositionally diverse liquid-phase bacterial membranes.^31^ Anisotropy was measured using the fluorophore, DPH, with a DPH:lipid ratio of 1:200. DPH was employed because it partitions primarily into the membrane, rather than into the aqueous environment,^32^ and distributes evenly throughout the membrane.^32^ Small, unilamellar vesicles were prepared as described in literature:^33,34^ chloroform-solubilized egg PG and DPH were mixed in a scintillation vial, spun-dry under nitrogen for 15 min, and then vacuum-dried overnight at 40 °C and 12 kPa. Dried films were rehydrated at 2.5 mg/mL in deionized water via 30 s of vortex mixing, 30 s of bath sonication, and 15 min of probe sonication at 20% amplitude using a UP200Ht from Hielscher Ultrasonics (Teltow, Germany). Metal debris from probe sonication was removed via centrifugation at 7800*g* for 30 min. Resulting liposomes were assessed via dynamic light scattering using a Malvern Zetasizer Ultra Red (Malvern, Worcestershire, UK) to have a *Z*-average diameter of 71.2 nm with a polydispersity index of 0.299, indicative of moderate polydispersity. Liposomes were diluted into cuvettes and allowed to equilibrate for 20 min before measurement.

Fluorescence anisotropy was measured using a FluoroMax Plus spectrofluorometer from Horiba, Ltd. (Kyoto, Japan), equipped with automatic polarizers. The fluorescence anisotropy, *r*, was calculated as

1 $$r=\frac{I_{VV}-G\cdot I_{VH}}{I_{VV}+2\cdot G\cdot I_{VH}},\mathrm{where}\;G=\frac{I_{HH}}{I_{HV}}$$

where *I*_*xx*_ is the intensity, and the first subscript indicates the polarization of the excitation at 359 nm, and the second subscript indicates the polarization of emission at 431 nm, *V* indicates vertical polarization, and *H* indicates horizontal polarization. *G* is the corrective “G-factor” inherent to the equipment under the given conditions. The dissolved polymer caused some emission; therefore, for each sample containing DPH, the intensity of an otherwise identical sample that lacked DPH was measured, and the intensities in eq 1 are the difference in intensity between the DPH and no-DPH samples.

### Zeta Potential

The cells were exposed to the relevant solution with or without PDADMAC. Zeta potential was measured with a Malvern Zetasizer Ultra Red (Malvern, Worcestershire, UK). Because that device also detects PDADMAC in solution, if PDADMAC were present in solution, then the result would be a weighted average of PDADMAC and PDADMAC-coated cells. To prevent this effect, cells that were exposed to PDADMAC were centrifuged and then resuspended in water twice before measurement.

### Statistical Analysis

All results show the mean and standard deviation of three independent experiments unless otherwise stated. *p*-values for Student’s *t*-tests were calculated for two-tailed heteroscedastic conditions. A *p*-value of <0.05 was the requirement to report a conclusion.

## Results and Discussion

### Salt Suppresses Antimicrobial Efficacy of PDADMAC

The effect of NaCl concentration on the efficacy of 4.6 × 10^5^ g/mol (∼2840 monomer) PDADMAC against E. coli in suspension was determined via a CFU assay that counts cells that can repeatedly divide to eventually form a visible colony. With no added salt, PDADMAC reduced the viable cell count by about 99.9% (3 logs) compared to water (Figure 1). The addition of 0.1 M NaCl caused no change, but as the concentration of salt increased, the efficacy of PDADMAC gradually diminished until there was no effect at 0.3 M NaCl.

**Figure 1:**
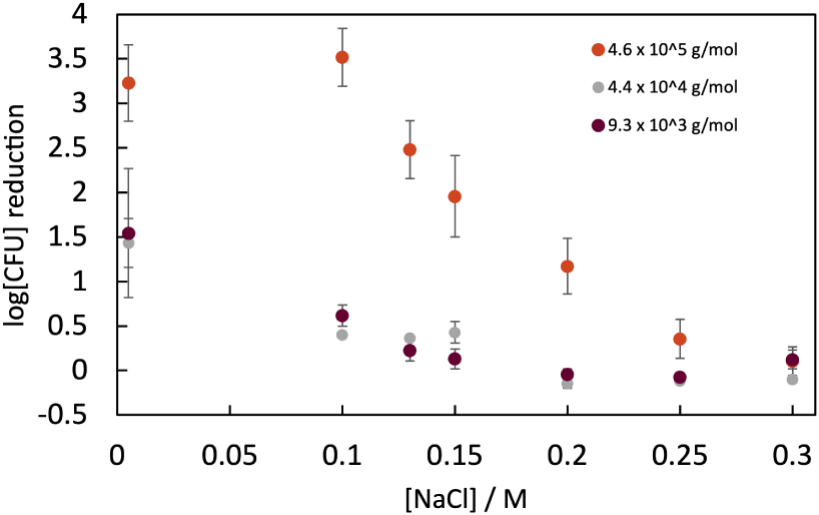
E. coli CFU reduction after 10 min in 10 μg/mL in a range of added NaCl concentrations. All results are compared to CFU counts in water using the following: log­[CFU] reduction = mean­[log_10_(CFU in water)] – mean­[log_10_(CFU in PDADMAC + NaCl soln)]. For 4.6 × 10^5^ g/mol PDADMAC, viability increases above 0.1 M NaCl, and PDADMAC is ineffectual above 0.3 M NaCl. A Student’s *t*-test comparing the 0 and 0.1 M data points for the 460,000 g/mol sample showed *p* = 0.2, indicating that the results are not significantly different. Note that the Cl^–^ ions from PDADMAC contribute only 6 × 10^–5^ M Cl^–^ so make a trivial contribution to the ionic strength. M stands for molarity.

PDADMAC with a much shorter molecular weight was less effective against E. coli. The efficacy of 9 × 10^3^ g/mol (∼57 monomers) and 4.4 × 10^4^ g/mol (∼280 monomers) PDADMAC was only about 2% of the PDADMAC that was the focus of this study (4 × 10^5^ g mol^–1^/2840 monomers). The polymer with 57 monomers is in the upper range of the number of monomers in antimicrobial polypeptides^35^ but with a much greater fraction of charged monomers. A similar trend of reduced toxicity to human lung carcinoma has been reported for polycations.^36^ The efficacy of these shorter molecules also diminished in the presence of 0.15 M or greater NaCl and therefore showed the same trend of reduced efficacy as the concentration of salt increased.

To investigate the reasons for the loss of PDADMAC efficacy in salt solutions, we examined single cells by fluorescence microscopy. 4.6 × 10^5^ g/mol PDADMAC was tagged with the fluorophore cy3, and we assumed that the adsorption of polymer was proportional to the intensity of emission. A cell membrane permeability indicator, Sytox Blue, was added. Once the cells become permeable, they are not viable and are usually considered to be “dead”. Further details of this approach are given in our previous work.^28^

In salt-free conditions, there was a high level of PDADMAC adsorption and subsequent cell permeation. These results are consistent with those of the CFU assay. In 0.15 M NaCl, far fewer E. coli cells were permeable than with no added salt (Figure 2). Moreover, the average amount of PDADMAC adsorbed on a cell was greatly reduced (Figure 3). The lack of adsorption and permeation was surprising, considering that CFU measurements indicated that only about 1% of cultured cells were viable (Figure 1) at that concentration: the cells are not dead but were unable to reproduce. At higher NaCl concentrations of 0.3 M, both techniques detected low levels of PDADMAC adsorption and efficacy. Likewise, exposure to 4.4 × 10^4^ g/mol PDADMAC produced some cells that are unable to reproduce but not permeable (not dead), but the number of these viable but not culturable cells was lower than for the 4.6 × 10^5^ g/mol PDADMAC. Because the lower molecular weight polymers produced weaker effects, in the remainder of this paper, we focus on the high molecular weight (4.6 × 10^5^ g/mol) PDADMAC. Similar effects of salt were also observed for S. aureus, a Gram-positive bacterium (Figures S1 and S2).

**Figure 2:**
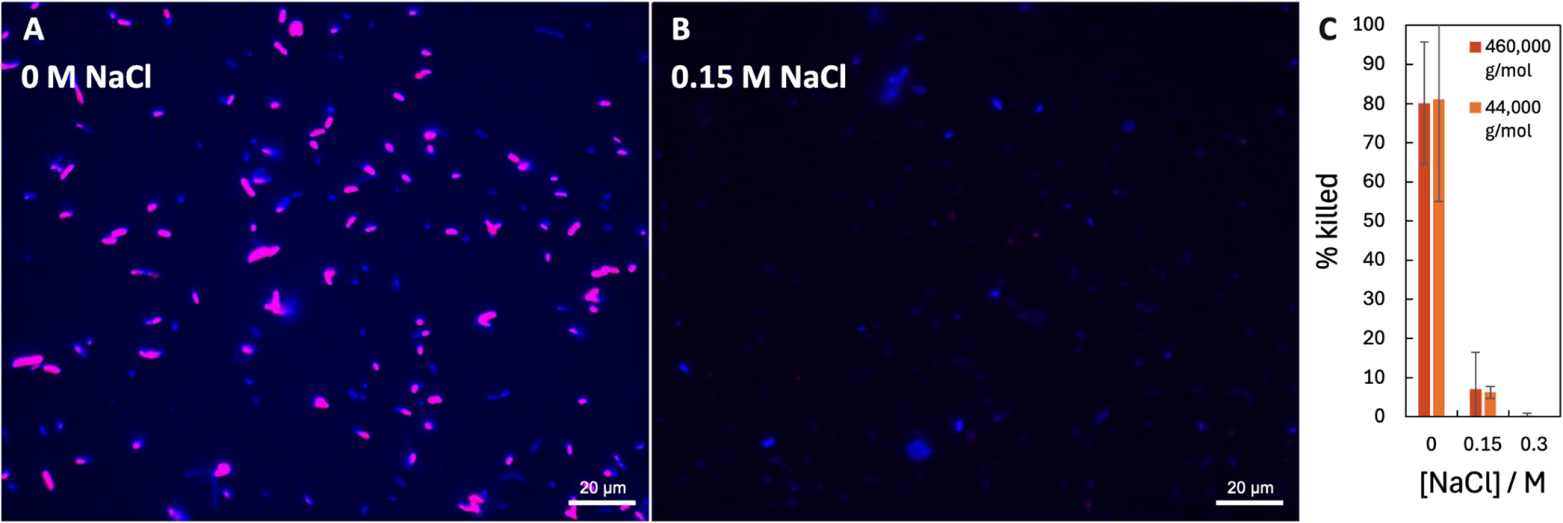
Adsorption to and killing of E. coli by 10 μg/mL PDADMAC. The fluorescence micrographs show results in 4.6 × 10^5^ g/mol PDADMAC in 0 M NaCl (A) and 0.15 M NaCl (B) after 60 min of flowing polymer solution. Without salt, there was strong adsorption to cells, indicated by PDADMAC fluorescence (blue color), and many cells became permeable, indicated by the red SYTOX Blue stain (red color). Cells where both adsorption and killing occurred are colored magenta. All dead cells are coated with polymer; therefore, there are no red-colored cells. With 0.15 M NaCl, there was a lower PDADMAC density, and there were no dead cells in the field of view. Part (C) shows the percentage of adsorbed bacteria killed in the flow cell (error bars are 95% confidence intervals) as a function of NaCl concentration for both 4.6 × 10^5^ and 4.4 × 10^4^ g/mol PDADMAC. No killing was detected for the 9.3 × 10^3^ g/mol PDADMAC sample. Killing was measured by a permeability stain. When the NaCl concentration was increased to 0.15 M, killing decreased by ∼11×.

**Figure 3:**
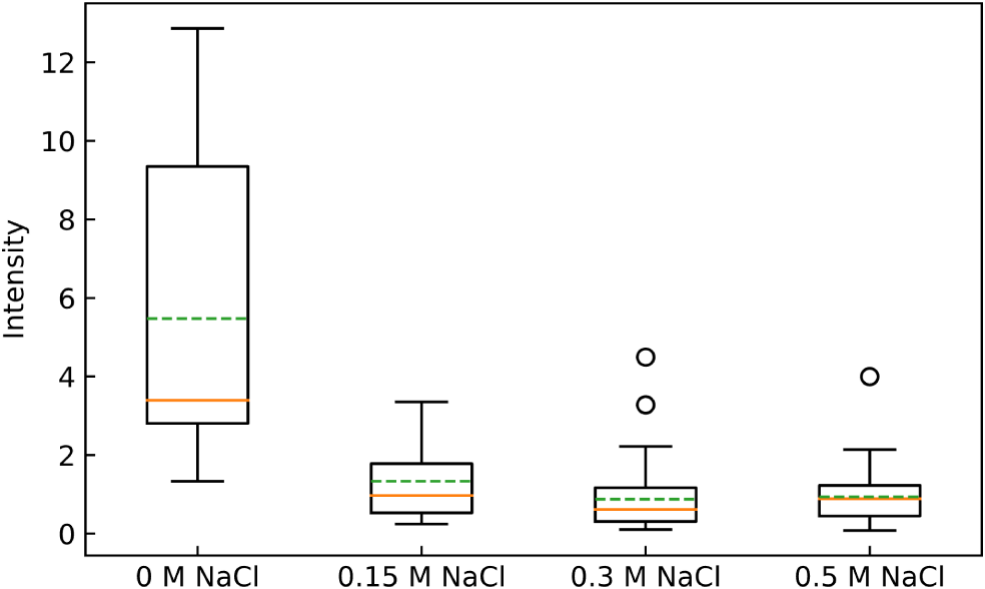
Maximum PDADMAC intensity (arbitrary units) measured for each individual E. coli cell with and without 0.15 M NaCl. Maximum intensity refers to the final equilibrium PDADMAC intensity after 60 min of flowing PDADMAC solution over the cells. The green dashed line is the mean, the orange solid line is the median, the box represents the interquartile range (IQR), and the whiskers represent the data range. Results are for three experiments under each of the conditions. About 100 individual bacteria were measured in each experiment. A *t*-test for a comparison of the mean intensity for 0 M NaCl and the other concentrations yielded *p* = 0.03, 0.03, and 0.02 for 0.15, 0.3, and 0.5 M NaCl, respectively.

### Lower Efficacy in NaCl Likely Arises Because Competitive Adsorption Reduces the Density of PDADMAC on the Cells

It is well-known that polyelectrolyte conformation is a function of electrolyte concentration: counterions bind to the polymer backbone and/or screen the charge such that the polymer adopts a less extended conformation in salt solution^5^ and this occurs in particular for PDADMAC.^29^ Adamczyk et al.^29^ showed that most of the decrease in length of PDADMAC occurs up to 0.01 M NaCl, and yet the antimicrobial efficacy was unchanged up to 0.1 M. We conclude that efficacy is not particularly sensitive to solution morphology. We propose instead that the main effect of salt on antimicrobial activity is the competitive adsorption by the salt. The zeta potential of our E. coli suspensions was −45 mV in water and −30 mV after being exposed to a 10 μg/mL PDADMAC solution (no salt) and then rinsed. Because PDADMAC is cationic and E. coli is net anionic, there is a strong electrostatic attraction driving the association of the polymer with the cells. In the presence of NaCl, ions compete with this association: PDADMA^+^ can bind to Cl^–^ instead of to the cell, and E. coli can bind to Na^+^ instead of to PDADMAC. Thus, competition for adsorption sites will result in a lower PDADMAC cell surface density.

Adsorption of PDADMAC is cooperative because the adsorption of one cation to the anions on the surface of E. coli brings neighboring cations into proximity with other anionic surface groups. To remove the effect of cooperativity of the adsorbate and focus on the effect of salt on the charging of E. coli, we also measured the effect of NaCl on the adsorption of a monovalent cationic dye, rhodamine 6G (R6G). Because rhodamine is monomeric, there is no Manning condensation on the rhodamine,^37^ and we expect that binding would simply depend on the availability of anionic sites on the E. coli. In contrast to PDADMAC, rhodamine is monomeric and rigid, so the addition of NaCl does not affect its conformation and has only a weak effect on the overall charge, so there is little effect of salt on the rhodamine itself. Therefore, by examining the effect of salt on the adsorption of rhodamine to E. coli, we can focus on the changes that salt brings to the surface of E. coli. Rhodamine 6G is hydrophobic, but we assume that the hydrophobic effect is a weaker function of salt concentration than the electrostatic binding.

We hypothesized that higher NaCl concentrations could result in less binding of R6G. To test this, a suspension of E. coli was centrifuged to separate the cells from the growth medium, and then resuspended in a series of solutions each containing R6G and a decreasing concentration of NaCl, from 0.5 to 0.01 M NaCl. The suspension was centrifuged, and the cells were separated from R6G in solution that did not adsorb, and then resuspended in a solution containing NaCl. The fluorescence emission of the washed cells was then measured. As shown in Figure 4, the emission from R6G was lower at higher NaCl concentrations. This loss of R6G binding is consistent with competition of Na^+^ with R6G for the anionic E. coli surface groups and Cl^–^ competing with the anionic E. coli surface groups for R6G. The displacement of R6G is consistent with the idea that the reduction of PDADMAC adsorption and antimicrobial efficacy in NaCl solutions is due to competitive adsorption of NaCl.

**Figure 4:**
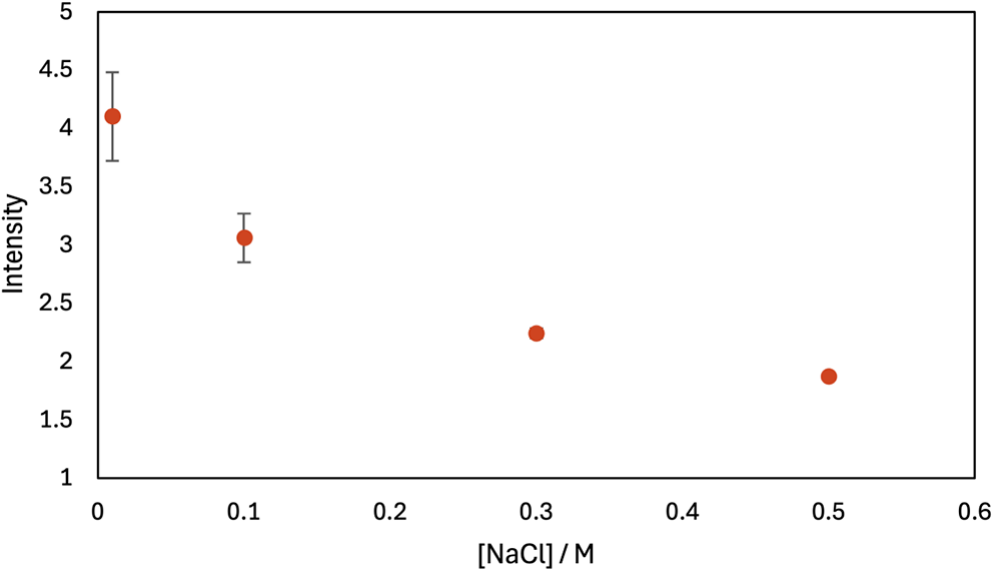
Fluorescence emission of E. coli cells suspended in a solution of 0.2 μm rhodamine 6G (R6G) plus various concentrations of NaCl was shown. R6G adsorption is greater at lower concentrations of NaCl. Intensity is total integrated fluorescence baselined with an E. coli suspension with no R6G. Intensity was measured after 1 min of mixing and 15 min of contact. The error bars represent the standard deviation of repeated measurements of R6G at each concentration. Some error bars are within the data points. Salt had a negligible effect on the fluorescence intensity of R6G in the absence of E. coli.

Previous research by our group showed that a threshold PDADMAC density must be achieved on the surface of a cell for it to be killed.^28^ Therefore, one explanation for the decrease in killing by PDADMAC in the presence of NaCl is simply that the lesser adsorption brought the PDADMAC adsorption below the cell’s threshold for death. Similar effects have been reported for cationic antimicrobial peptides. Matsuzaki et al. showed that the addition of salt decreased antimicrobial peptide binding to liposomes and reduced subsequent leakage.^38^ To the best of our knowledge, a similar result has not been observed previously with a synthetic polycation or with bacteria.

At this point, there is an unresolved question as to why the cells in PDADMAC and 0.15 M NaCl are not able to reproduce, as evidenced by the CFU measurements, even though light microscopy shows intact cells, and the permeability stain shows that the cell envelope is intact. 0.15 M NaCl alone does not affect E. coli viability.^39^ Part of the answer lies in Figure 3, which indicates that, although adsorption of PDADMAC is very low, it is not absent. So, this smaller, nonlethal, density of polymer is likely acting to reduce the ability of E. coli to divide. This idea is examined in the following sections.

### PDADMAC Decreases the Fluidity of a Model Cell Membrane

We used model membranes in the form of small, unilamellar liposomes to understand the impact of PDADMAC on the fluidity of bacterial cell membranes; such models have been used previously^40^ and simplify interpretation. Egg-derived phosphatidylglycerol (egg PG) was selected because both the liposomes it forms and E. coli are negatively charged and in the liquid phase. DPH was incorporated into the liposomes to probe their membrane fluidity, which was assessed via DPH fluorescence anisotropy. We followed the work of others by attributing an increase in DPH anisotropy to an increase in rigidity of the lipid bilayer.^40^ Our results show that the presence of PDADMAC in solution increases anisotropy (Figure 5) and therefore increases the rigidity of the lipid membrane. We rationalize the increase in rigidity to polycation molecules bridging between anionic lipid headgroups, which reduces the lateral fluidity of the headgroups and, in turn, the flexibility of the chains that solvate the DPH.

**Figure 5:**
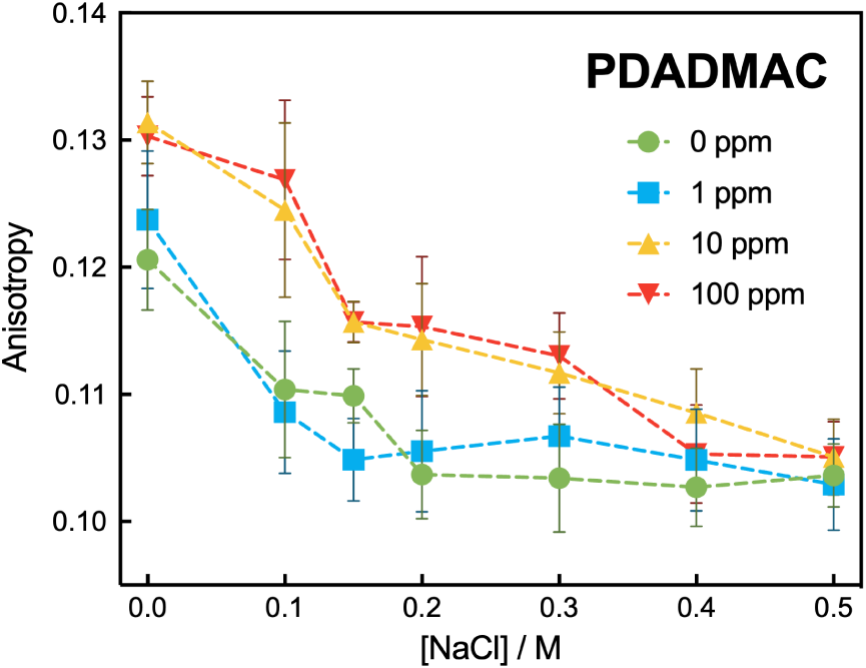
Fluorescence anisotropy of DPH in egg PG model membranes as a function of NaCl concentration with varying concentrations of PDADMAC following 20 min of equilibration. Symbols represent the average of three independent experiments, the error bars represent standard deviation, and the lines are guides to the eye. PDADMAC increases the anisotropy, which we interpret as an increase in rigidity of the lipid membrane, and this effect diminishes with greater salt concentration.

The goal here is to determine the effect of the salt. The change in anisotropy as a function of NaCl concentration with no polymer (Figure 5) was used as a control. NaCl decreases the anisotropy and therefore decreases the rigidity. Prior work showed that salt increases the spacing of the lipid headgroups^41^ by lowering the activity of protons in solution that causes an increase in charge density. The increased spacing of the headgroups should allow more chain fluidity. In the absence of PDADMAC, the main change in anisotropy occurs for concentrations up to 0.1 M. Considering now liposomes in a solution of 10 μg/mL PDADMAC and salt, the anisotropy remains high at 0.1 M salt and then drops between 0.1 and 0.15 M salt. This is the same range over which a decrease in adsorption and killing was observed, so there is a correlation between rigidification, loss of adsorption, and loss of ability of the bacteria to form colonies. We rationalize the loss of anisotropy (rigidity) upon addition of salt as follows: the Na^+^ ions compete with the quaternary ammonium ions which decreases polymer adsorption, decreasing the amount of ionic bridging by PDADMAC that caused the rigidity. To summarize the results here and earlier, salt reduces the adsorption of PDADMAC. This decreases the rigidifying effect of PDADMAC and prevents the PDADMAC from killing the bacteria.

### A Coating of PDADMAC Formed in 0.15 M Ionic Strength Hinders Cell Growth and Division

Earlier, we observed that 0.15 M NaCl and PDADMAC do not kill E. coli, but they do greatly reduce the ability of E. coli to form colonies. Here, we sought to reconcile these measurements through a microscopic study of cell growth and division. For the microscopy experiments described earlier in this work, the lack of nutrition in the solution (medium) prevented cell growth. For experiments in this section, the medium was changed to a minimal salt medium, m9, which is used to cultivate E. coli.^42^ Standard m9 was diluted by 50% such that the final concentrations were 3 g/L KH_2_PO_4_, 0.5 g/L NaCl, 6.78 g/L Na_2_HPO_4_, and 1 g/L NH_4_Cl, which corresponds to an ionic strength, *I*, of 0.15 M in keeping with conditions used earlier in this work. Glucose was added to provide a carbon and Gibbs energy source for growth. SYTOX Blue was added to the growth medium as a permeability indicator. Control experiments showed that cells grew in 50% m9 without PDADMAC and that PDADMAC inhibited reproduction (Figure S3).

The cell length was monitored by microscopy for 1 h in growth media, with and without PDADMAC, and was measured using ImageJ software. As shown in Figure 6, the addition of PDADMAC to growth media greatly reduced cell growth. In the absence of PDADMAC, the average increase in length was 0.36 μm, whereas with PDADMAC, it was 0.06 μm; in the absence of PDADMAC, more than 75% of the cells grew by more than 0.2 μm, whereas in PDADMAC, fewer than 25% grew by this length. Additionally, no cell death occurred, confirming that PDADMAC at *I* = 0.15 M is no longer bactericidal, but bacteriostatic (halts growth).

**Figure 6:**
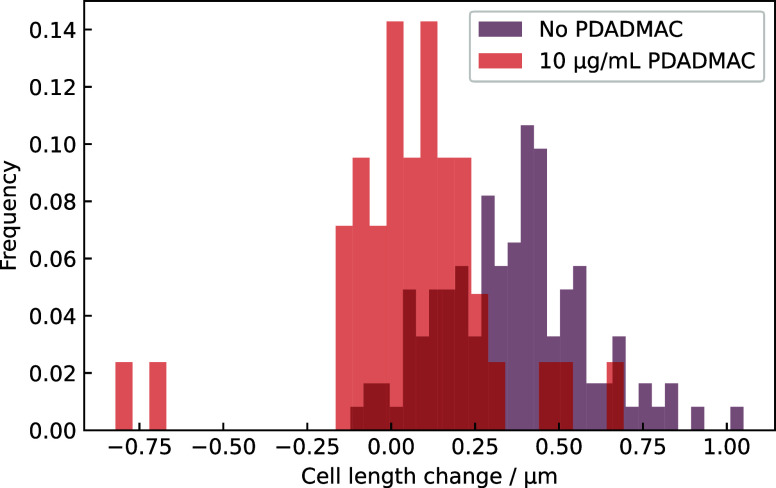
Histogram of change in E. coli cell length in minimal growth medium (ionic strength = 0.15 M) after 60 min in 10 μg/mL PDADMAC. The length of each cell in the microscope field of view was measured initially and was measured again either when it divided, or at 60 min, whichever occurred first. For example, a cell that began with 0.6 μm length and was 1.0 μm in length when it divided after 30 min was recorded as 0.4 μm change in length. Cells in the absence of PDADMAC start at different times in the cell cycle; therefore, they grow by different lengths before they divide. Cells do not grow in 10 μg/mL PDADMAC at *I* = 0.15 M. Mann–Whitney *U* test comparison of the two populations gives *p* = 3 × 10^–22^ for the comparison of the populations.

At this stage, we made the conjecture that the E. coli cells in PDADMAC and *I* = 0.15 M were alive but simply physically restrained from growth by a layer of polymer. If true, then removal of the PDADMAC coating would allow the cells to grow. To test this, Dey-Engley (DE) broth was flowed over the cells. DE broth was formulated to halt the effect of antimicrobials, including polycations,^42^ and we guessed that it might wash PDADMAC from the cells. DE broth contains a large mixture of ingredients, and the mechanism of action for each ingredient is not known. We flowed PDADMAC and 0.15 M NaCl over E. coli for 60 min to coat cells in PDADMAC and then flowed DE broth (flow rate increased to 20 mL/h) over the cells. Fluorescence emission showed that the PDADMAC was removed over a period of 60 min, and most importantly, the cells commenced growth immediately after the removal. Therefore, PDADMAC was restraining growth rather than causing irreversible damage. Data for one cell (Figure 7) shows that it grew and divided 10 times over about 8 h subsequent to PDADMAC removal. Regrowth after removal of PDADMAC was typical, but a few cells did not grow because their affinity for PDADMAC was so high that it could not be removed, or, in very few cases, the cells did not grow even when PDADMAC was removed. Presumably, the latter cells were dead. After demonstrating that DE broth can remove PDADMAC, we determined that PDADMAC absorbed in *I* = 0.15 M could also be removed simply by long-term washing with water.

**Figure 7:**
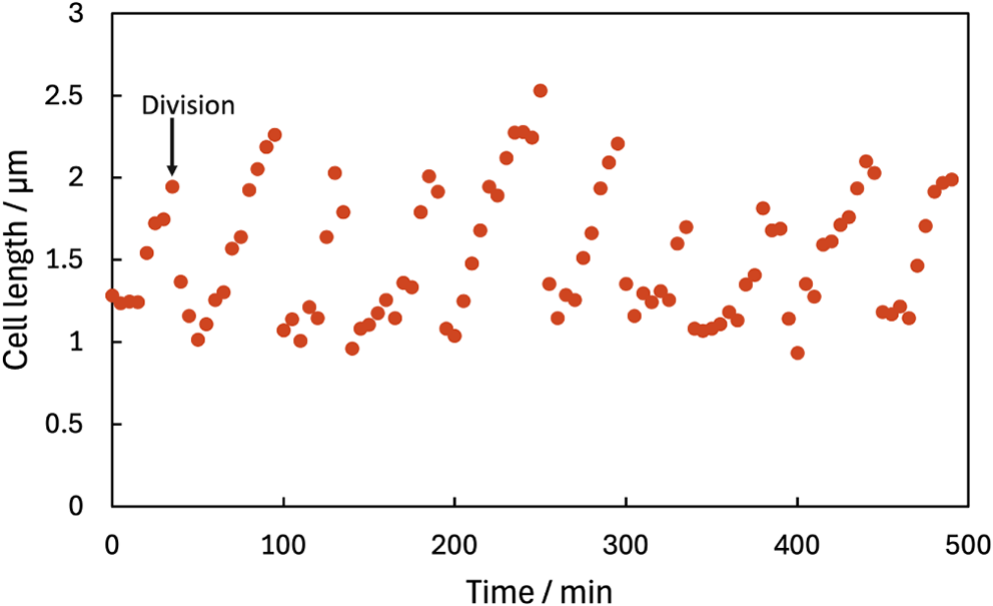
Growth of an individual E. coli cell after PDADMAC was rinsed off the cells. Measurement was from optical microscopy images. Cells were exposed to solution containing both 10 μg/mL PDADMAC and 0.15 M NaCl for 60 min; then, the flow was switched to DE broth until PDADMAC was removed, approximately 100 min into the experiment. Time = 0 in this figure corresponds approximately to the time when the PDADMAC was removed. The cell was static until PDADMAC was removed, then divided once every ∼50 min. The first division is indicated by the arrow.

Combining results from this and the previous section, we conclude that PDADMAC adsorbed in 0.15 M NaCl or *I* = 0.15 M binds to the surface of E. coli and physically prevents the cell wall from undergoing the necessary expansion for growth and reproduction. We write “physically prevents” because the results from anisotropy measurements showed that liposomes become rigidified. The PDADMAC with salt does not cause irreversible chemical damage, as does, for example, PDADMAC without salt (or bleach, etc.). The PDADMAC deposited from 0.15 M NaCl did not kill the cells, and when it was removed, the cells were able to grow in length and reproduce, confirming that cell growth is only prevented when PDADMAC is actively coating the cell. PDADMAC plus salt can therefore be used to hold the cells in a static condition. Previous work has demonstrated other conditions where cells were maintained in static conditions.^43–45^

### E. coli Cell Dimensions Decrease after Exposure to PDADMAC in Water, But Not in NaCl Solution

When E. coli is exposed to PDADMAC-only solution, the cell length decreased (Figure 8), whereas cells in PDADMAC plus 0.15 M NaCl maintained the same length. Cells in PDADMAC decreased in length regardless of whether they were alive or dead, and shrinkage occurred only after adsorption of the polymer but prior to cell permeabilization; it is not an effect of permeation. Adsorption typically occurred after 5 min, the reduction in length occurred after 10 min, and permeation was recorded after 20 min. We interpret this shrinkage as due to bridging of the ionic groups on the surface of E. coli. Discharge of the PDADMAC groups through binding with cell surface groups should reduce the repulsion between quaternary ammonium groups and decrease the extended length of PDADMAC. The contraction of the bound PDADMAC would drag cell surface groups and cause damage. Damage was indicated by the permeation dye. On the time scale of our measurements (<2 h), no cell rupture was observed on the scale allowed by the ∼300 nm resolution of light microscopy, but this is a large scale by the standards of a bacterial cell. Evidently, being shrunk by PDADMAC is a terminal experience for E. coli, and this may be part of the mechanism of cell death by PDADMAC. All cells that shrank in length eventually died.

**Figure 8:**
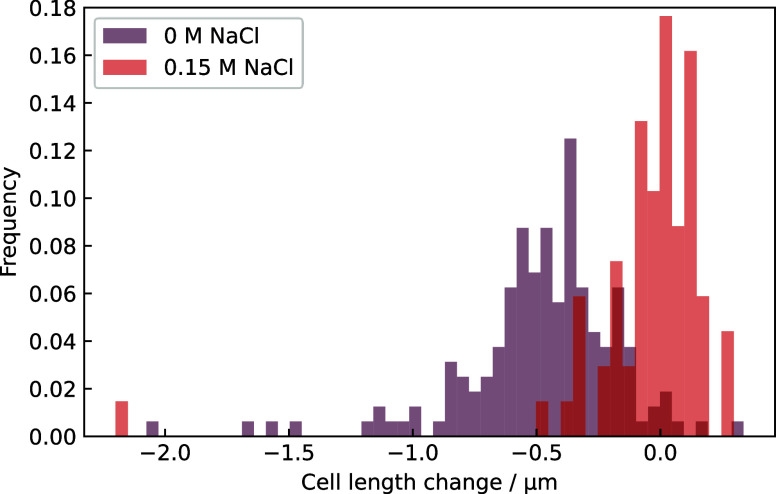
Histogram of cell length change during exposure to 10 μg/mL of PDADMAC for ∼1 h for two concentrations of salt. The average decrease in length in 0 M NaCl was 0.48 μm and for 0.15 M NaCl was 0.05 μm. Each bacterium in the plot was manually identified and measured using ImageJ at the beginning and end of the experiment. PDADMAC typically shrinks cells, but in the presence of 0.15 M NaCl there is no shrinkage on average, even though polymer still adsorbs. *p* = 1.64 × 10^–24^ for the effect of salt using Mann–Whitney U test.

In 0.15 M NaCl, we rationalize the lack of shrinkage as follows: the polycation has a less extended conformation and is more flexible, and our results are consistent with fewer binding points to the cell. Therefore, in salt, the polymer exerts a weaker contractile pull on the cell surface.

Prior work has also demonstrated polymers that reduce the bacterial cell size. For instance, Pilizota et al. showed that dextran (a polysaccharide) caused E. coli to shrink in size but attributed this to osmotic pressure effects.^46^ For PDADMAC, the concentration is too low to have an osmotic effect on the bulk solution, and we directly observe a correlation between the timing of adsorption and shrinkage.

### A Coating of PDADMAC Formed in Water is Not Always Terminal for All Cells

We investigated whether it was also possible to remove PDADMAC deposited from salt-free solutions onto E. coli. A previous paper by our group showed that there was a lag time between the adsorption of PDADMAC and the permeation of the cell.^28^ The question here was whether a cell could be saved from death by removal of the PDADMAC during this lag time. To test this, PDADMAC with no salt was flowed over the cells for 20 min, which coated the cells in a lethal density of polymer, and then the flow was switched to DE broth to remove the adsorbed polymer. After this procedure, about 20% of cells were permeabilized and, not surprisingly, never regrew. For the cells that did not become permeable, roughly 75% regrew, but 25% never did. We conclude that it is possible to save some cells by removing the polymer before the cell became permeable. This suggested that there is a slow rearrangement of PDADMAC and the cell surface groups that occurs after adsorption and leads to cell death.

## Conclusions

Over 99.9% of cells are killed by 4 × 10^5^ g mol^–1^ PDADMAC adsorbed from water, but when adsorbed from 0.15 M NaCl, PDADMAC no longer kills E. coli; it only stops cell growth. Likewise, in 0.15 M salt, PDADMAC did not kill S. aureus; therefore, similar effects are observed for Gram-negative and Gram-positive bacteria. The lack of killing by PDADMAC in 0.15 M NaCl may be due to a number of effects, including less adsorption, reduced membrane rigidification, and less shrinkage of the cell. The last two effects are probably consequences of the lesser adsorption and a less extended conformation of PDADMAC in salt.

PDADMAC still adsorbs to E. coli from 0.15 M salt, and once cells are coated in a sufficiently high density of PDADMAC, they no longer grow in length. Since normal bacterial reproduction occurs via growth and then division, this is consistent with the lack of formation of colonies observed via CFU measurements. The adsorption and bacteriostatic effect in 0.15 M ionic strength is reversible: cells regrow when PDADMAC is removed from the cell surface, and a source of nutrition is provided. Because the bacteriostatic effect of PDADMAC in salt is reversible, we conclude that growth is suppressed by the polymer physically binding anionic groups together on the surface of the cell such that the cell cannot grow, rather than due to the formation of pores or other permanent modifications. This conclusion is supported by measurements of fluorescence anisotropy on bacterial membrane mimics (anionic liposomes), demonstrating that PDADMAC adsorption increases membrane rigidity, but less rigidification occurs for PDADMAC that is adsorbed from a 0.15 M NaCl solution.

If adsorbed PDADMAC from salt-free solutions is removed quickly by rinsing, then cell death can be prevented, even when a dense coating of PDADMAC covers the cells. This suggests that the mechanism for cell death is not merely adsorption of polymer but also requires a slow (minutes) rearrangement of the polymer that damages the cell, prevents release of the polymer, and is not reversible.

We also found that lower molecular weight PDADMACs (57 or 280 monomers) were much less effective antimicrobials, with efficacy that was only about 2% of the PDADMAC that was the focus of this study (4 × 10^5^ g mol^–1^/2840 monomers). These shorter molecules also had reduced efficacy in the presence of 0.15 M or greater NaCl.

## Supplementary Material



## Notes

S. aureus CFU reduction after 10 min in a range of added NaCl concentrations and fluorescence microscopy of individual cells; E. coli growth in 50% m9 minimal salts media (PDF)
