# Supplementary material for: Effect of Salt on Synthetic Cationic Antimicrobial Polymer–Cell Interactions
Source: Biomacromolecules. 2025 May 19;26(6):3322–30. doi: 10.1021/acs.biomac.4c01706 (PMC12152946; doi:10.1021/acs.biomac.4c01706)
Supplement: Supplementary file 1 [file bm4c01706_si_001.pdf]

Supporting information

Effect of salt on synthetic cationic antimicrobial polymer-cell interactions

Zachary Benmamoun[1], Thomas Kinard[1], Prem Chandar[2], Joe Jankolovits[2], and William A. Ducker[1.]

[1.] Department of Chemical Engineering, Virginia Tech, Blacksburg, VA, 24060, USA

[2.] Unilever Research & Development, Trumbull, CT 06611, USA

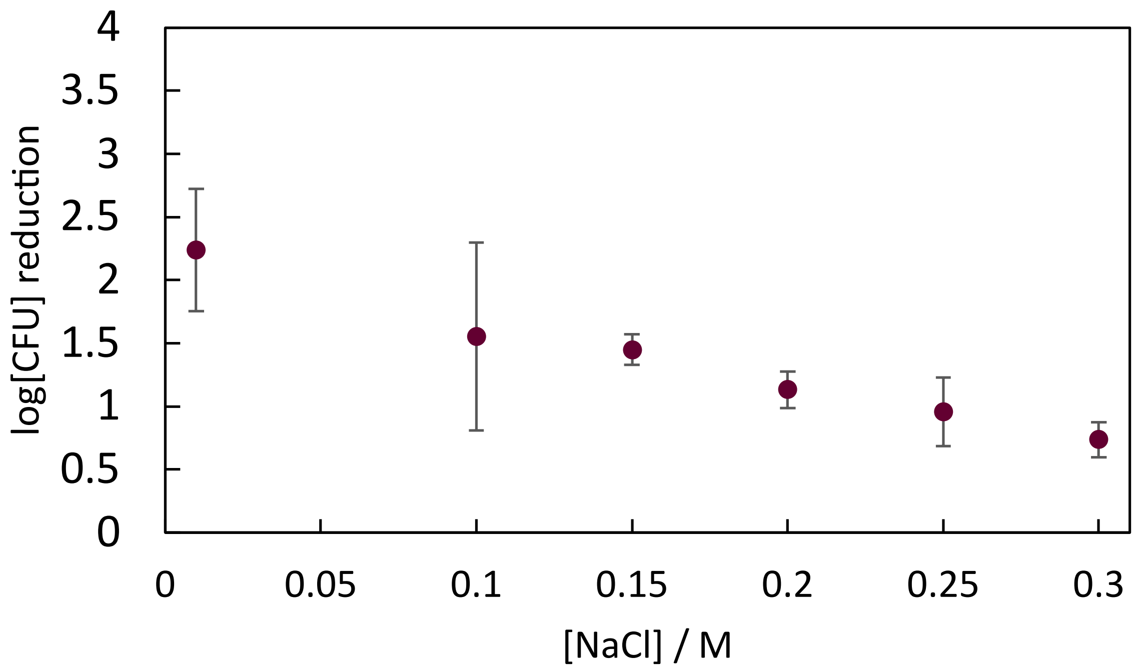

Figure S1. *S. aureus* CFU reduction after 10 min. in 10 µg/mL 4.6 x 10^5 g/mol PDADMAC in a range of added NaCl concentrations. These results have a similar trend to those in Fig. 1 for *Escherichia coli* (*E. coli*), but the range of the reductions is smaller.

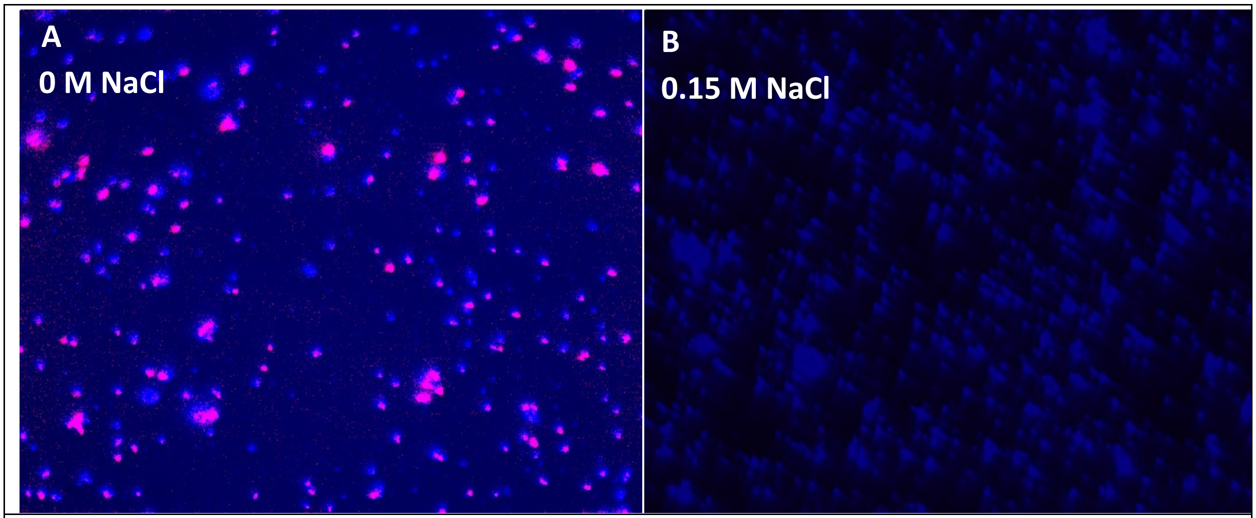

Figure S2. Adsorption to and killing of *S. aureus* by 10 µg/mL 4.6 x 10^5 g/mol PDADMAC in (A) 0 M NaCl and (B) 0.15 M NaCl after 60 minutes of flowing polymer over the cells. Fluorescence microscopy indicates PDADMAC adsorption (blue color) and cell permeation (red color). Cells where both adsorption and permeation occurred are colored magenta. Without salt there was strong adsorption of PDADMAC to cells and many cells became permeable; in 0.15 M NaCl there were no dead cells in the field of view. This response of *S. aureus* is similar to that of *E. coli.*

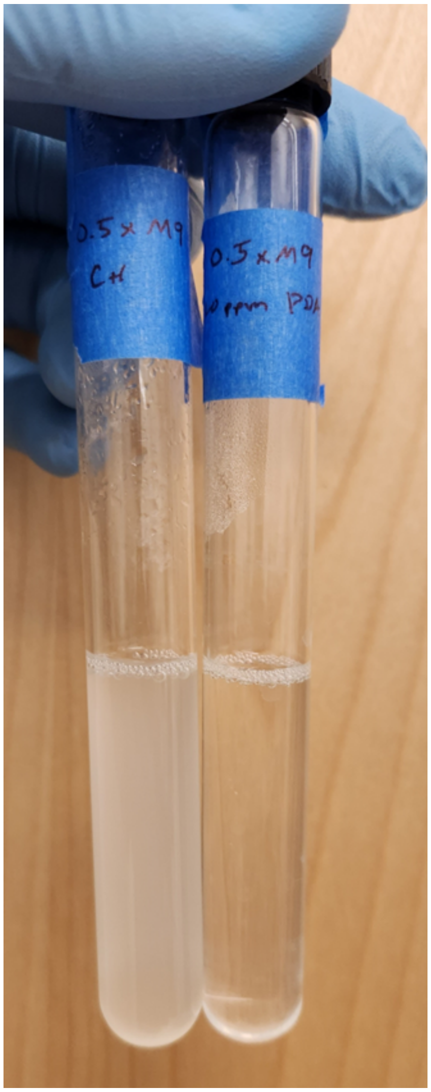

Figure S3. *E. coli* growth in 50% m9 minimal salts media. The left tube shows solution with zero PDADMAC and the right tube shows 10 µg/mL PDADMAC. The turbidity in the absence of PDADMAC shows growth; PDADMAC reduces growth.
